# Improvement in oral health related quality of life among the elderly: a randomized controlled trial

**DOI:** 10.1186/s13030-019-0170-3

**Published:** 2019-12-05

**Authors:** Elham Shokouhi, Hashem Mohamadian, Fatemeh Babadi, Bahman Cheraghian, Marzieh Araban

**Affiliations:** 1 Department of Health Education and Promotion, Public Health School, Ahvaz Jundishapur University of Medical Sciences, Ahvaz, Iran; 2 Department of Health Education and Promotion , Public Health School, Ahvaz Jundishapur University of Medical Sciences, Ahvaz, Iran; 3 Department of Oral and Maxillofacial Medicine, Faculty of Dentistry, Ahvaz Jundishapur University of Medical Sciences, Ahvaz, Iran; 4 Department of Biostatistics and Epidemiology, Public Health School, Ahvaz Jundishapur University of Medical Sciences, Ahvaz, Iran; 5 Department of Health Education and Promotion, Social Determinants of Health Research center, Public Health School, Ahvaz Jundishapur University of Medical Sciences, Ahvaz, Iran

**Keywords:** Adult learning theory, Oral health related quality of life, Elderly, Health education

## Abstract

**Background:**

The present study was conducted to determine the impact of educational intervention based on adult learning theory on oral health related quality of life of the elderly.

**Materials and methods:**

This study (IRCT20120910010804N13) was performed with 92 elderly patients referred to the dental clinic of Ahvaz Jundishapur University of medical sciences. Participants were randomly divided into experimental and control groups. The data were gathered by a questionnaire with demographic variables, variables of oral health related quality of the elderly, and variables for assessing the effectiveness of adult learning theory. Following pre-test, educational programs were conducted for the interventional group. After 1 month, the questionnaire was again administered to both groups. Next, the results of pre-test and post-test were analyzed using SPSS-23 at a significance level of 0.05.

**Results:**

Educational intervention was significant in terms of overall oral health related quality of life and the overall effectiveness score of adult learning theory (*P* < 0.001). There was a significant difference between the two groups in terms of the mean change score of three physical, psychosocial, and pain dimensions following the educational intervention (*P* < 0.001).

**Conclusion:**

Education based on adult learning theory is recommended for improving oral health related quality of life among the elderly.

**Trial registration:**

Iranian Registry of Clinical Trials, IRCT20120910010804N13. Registered on 2018-12-16.

https://www.irct.ir/trial/35239

## Background

The increase in the elderly population due to the increase in life expectancy [1] requires adequate attention to the different needs of this age group [2] that subsequently influences their quality of life, which is considered to be a social development measure [3, 4]. According to studies conducted in different countries, high aged populations are a global phenomenon; here, in Iran like other developing countries, The aged society has become a major health issue as aging increases the prevalence of illness and disabilities [5–7].

Old age is associated with some physiological changes, low appetite, nutritional problems, oral and dental problems such as loss of teeth, and difficulty in swallowing and chewing food, followed by a variety of illnesses [8]. In addition to the natural changes occurring in the body system with age and aging, chronic illnesses increase as age rises. The prevalence of chronic diseases is augmented by a decrease in the normal functioning of the aging body over time, loss of function, dependence on others, isolation, disability, and reduction in the quality of life and satisfaction of the elderly [5, 9, 10]. The phenomenon of aging increases the burden of all chronic diseases, including oral diseases [11, 12]. Oral health problems have social, economic and psychological consequences affecting the life quality of the elderly [2]. Although oral health status was historically assessed by epidemiological surveys using objective clinical indicators, currently, specific measures of oral health-related quality of life (OHRQoL) are also used [13, 14]. Quality of life is one of the biggest health goals for improving the health of the elderly [15]. Such health goals include a multidimensional concept that includes physical, psychological, social, mental, and disease-related symptoms or changes in treatment [9, 11]. The oral health related quality of life is the individual’s assessment of how their mental, psychological, and social status are affected by their oral health [16]. The findings of a study done by Mack (2005) revealed that the impact of reducing the number of teeth without replacing them on QoL was as much as cancer and kidney diseases [17]. Cornejo’s study (2013) on the oral health related quality of life in the elderly in Barcelona showed that 68.1% of women and 64.3% of men had poor oral health related quality of life [18]. Kidd (2017), in her systematic review study, indicated that self-management interventions improved health-related quality of life in 6 out of 7 studies [19]. In order to enhance the oral health related quality of life of the elderly, it is essential for society to create opportunities to educate this age group. Beneficial effects of health promotion by educational programs are documented in several studies. Research further indicates that a health education program plays a crucial role in increasing healthy life span and life quality [20]. Studies have shown that education will have a significant impact on the health behavior of individuals [21, 22]. It has been reported that theory-driven intervention is more effective in changing behavior than are health outcome interventions [23]. One such theory is the adult learning theory, which refers to an organized process for raising the awareness, cognition, and skills of adults in order to be able to move towards excellence and evolution. The experience of people in the learning process has an important role in this theory. In addition to experience, adults’ desire to learn without any compulsion (unlike children) is one of the strengths and reasons for using this theory [24]. According to the latest population and housing census in 2016, the relative distribution of 65 year-old and older people in Khuzestan, Iran was 4.4%, which shows a 3% increase in the elderly population in comparison with the 2011 census data [25]. Although several studies have been conducted on the oral health related quality of life of the elderly in different countries, few studies have investigated the effect of an educational intervention on the oral health related quality of life of the elderly. Accordingly, the aim of the present research was to evaluate the effectiveness of an educational intervention on the oral health related quality of life of the elderly.

## Methods

### Design

This randomized controlled trial study was conducted from 31 June to November 2017 in the dental clinic of Ahvaz Jundishapur University of Medical Sciences, with all participants aged 60 years or older, according to the United Nations scale of aging [26].

### Sample

Inclusion criteria were fluency in Persian, informed consent to participate in the study, good psychological and mental status according to the clinician’s diagnosis, minimum score on the short cognitive performance test (> 6) [27], and literacy. Exclusion criteria were absence from training sessions, death, use of psychoactive drugs, blood pressure of more than 140/80 mmHg, use of anticonvulsant and antidepressants during the study, smoking and alcohol consumption during the study, radiotherapy history, the presence of any oral ulcer including oral aphthous, fungal ulcer, benign and malignant lesions as diagnosed by a dentist, complete dentition, and certain systemic diseases. According to the opinions of dental experts on oral diseases, these conditions affect the quality of life of individuals, therefore, participants with these criteria were excluded from the study.

In order to determine the sample size, the comparison of two-means formula was used in which *α* =0.05, *β*= 0.1, and, based on the results of previous studies [28], X1 = 46.59, X2 = 58.94, S1 = 17.65, and S2 = 12.79. The initial sample size was two groups of 34. Considering the probability of 25% of the subjects dropping out of the study, the final sample size was equal to two groups of 46 persons (92 individuals in total).

$$ {n}_1={n}_2=\frac{\left({S}_1^2+{S}_2^2\right){\left({Z}_{1\hbox{-} \frac{a}{2}}+{Z}_{1\hbox{-} \beta}\right)}^2}{{\left({\overline{X}}_1\hbox{-} {\overline{X}}_2\right)}^2} $$

### Procedures

Prior to performing the pre-test and after an explaination of the design and purpose of the study and how it was to be conducted, written informed consent was obtained from each subject.

In order to determine which subjects had the ability to respond to the researcher psychologically and mentally, a brief Persian version of a mental test was given by a short cognitive performance test. The Short Cognitive Performance Test (Syndrom-Kurztest, SKT) was originally developed and validated in Germany by Erzigkeit in 1991. Finally, the subjects who scored a minimum of (> 6) were included in the study [29, 30].

Subjects were assigned to the study groups (intervention and control) via block randomization using six blocks, and WinPepi11.0 software, which generates random groups. In each block, three subjects from the control group and three subjects from the intervention group were randomly included. Finally, 16 blocks were used. The sampling started from the first option of block 1 until option 2 of block 16, then continued until it reached 92. The randomization of the participants was carried out by a statistical consultant. The pre-test for both groups was performed by completing an unnamed, pre-coded questionnaire, then it was returned to the researcher. Collecting the 92 samples’ pre-tests finished at the end of 2017 July the end of July 2017.

### Intervention

The intervention was based on adult learning theory, first introduced by Malcolm Knowles at the beginning of the 1970s. In this theory, experience, critical thinking, and logical dialogue are the three main issues. In adult education, the desire to learn is more important than learning itself, as adults are forced to learn or attend classes. The desire to learn of adults may be derived from a serious interest, though temporarily, in certain issues and the need for knowledge and skill to solve a problem [31].

After the pre-test, a training program comprised of a combination of in-person training (individual training and group discussion) and non-attendance training (sending educational messages) was prepared and submitted to the intervention group as follows:

A part of the in-person training was performed immediately following the completion of the pre-test questionnaire in the form of 15 min of individual training based on motivational interviewing methods. Educational tools such as a researcher-made booklet were developed, and a dental modulate was used to improve individual education.

#### Face-to-face (individual) training using motivational interviewing

Adult learning theory emphasizes the creation of a learning environment where there is always interaction and cooperation between the tutor and learner, and there exists the opportunity to learn actively through the exchange of information and comments between the trainer and instructor in individual training methods [32], hence the selection of educational method as one of the educational methods in this research.

The purpose of doing the individual training method through motivational interviewing is to increase the motivation of the elderly to take care of their oral health and to prevent oral problems, in addition to providing educational points and materials related to their oral health. The motivational interview with the experimental group was conducted as follows:

We tried to empathize with the participants and show that their oral health related quality of life was very important. First, to encourage them to talk, they were asked an open-ended question about the importance of oral health to the elderly. While listening to the responses, the participants’ statements were summarized for confirmation. At this stage, the researcher tried to fathom the emotions and problems of the participants about the issue under discussion. After that, an attempt was made to change the language of conversation in order to change the behavior, as follows: “It is very important that you have decided to deal with your teeth discomfort despite financial problems.” In the final stage, by expressing sentences such as “there are very simple ways to protect teeth which I will teach you”, an agreement to change behavior between educator (one therapist) and participants was created. To provide the subjects with useful information about oral health, a researcher-made booklet was developed and approved by dental practitioners and health education experts. It is to be noted that in order to provide more effective content, the booklet was prepared in color. Furthermore, a dental moulage was also used to teach the correct way of brushing and proper use of dental floss. Moulage was employed because, based on adult learning theory, adults learn better through doing [32]. Therefore, after the completion of training, each subject was asked to do what they had learned to the best of their ability, a step conducted by the researcher. One therapist (health education specialist) attended a face-to-face interview.

### E-learning (sending educational messages)

Media interventions via sending educational messages are effective in promoting productivity and increasing interventions [33]. Remote training was done by sending educational messages concerning the importance of oral health. Three short text messages were designed and approved by health education, health promotion, and oral and dental specialists. The messages were about the importance of preventing oral health problems, the basis for the prevention of oral health problems, and regular referral to the dentist. One week following the face-to-face training, educational messages were sent once a week for 3 weeks. Delivery of a report ensured that the participants received the text messages. An example of the text messages is: “Prevention of oral and dental diseases is much easier and less costly than treatment”. The training continued for one session via a group discussion methodology, and 1 week following the completion of sending the messages a group discussion was conducted.

## Group discussion

A one-hour group discussion was conducted for the subjects to share their experiences on issues related to their oral health, where the participants were asked to talk about their oral health problems and to state how these problems had affected their personal lives and social relationships. To conduct group discussions, the participants were divided into two groups, one with 22 participants and the other with 21, with each group guided by 1–2 dentists and experts in health education (researcher). Adult learning theory states that adults learn from their past experiences [34], and in group discussion methods, individuals can equally participate and share their experiences, hence the fact that this method of training was selected and implemented.

After completing the educational intervention, the subjects were followed up for 1 month, after which the questionnaire was administered again to both groups, giving a pre and post test time difference of 2 months. It is to be noted that the control group did not receive any of these training programs during the study, only receiving routine care. Also, in order to reduce the probability of information bias, the person evaluating the results (post-test) of the educational group was blinded to the participants’ group assignment.

### Outcome measures

The data collection tool comprised demographic characteristics of the participants, questions about the oral-health related life quality, and questions about the effectiveness of adult learning theory.

#### Demographic sheet

The first part of the questionnaire included variables such as age, sex, marital status, educational level, and occupation. For this purpose, a checklist about the history of patients, designed by the faculty of dentistry, was employed.

#### Geriatric oral health assessment index

The second part of the questionnaire consisted of 12 questions about the oral health status of the elderly over the past 3 months. Because the intervention lasted for 2 months, we asked the participants to report the status of their oral health over the past 2 months to prevent a time overlap. This part of the questionnaire was derived from the standard questionnaire on geriatric oral health assessment index (GOHAI) of the elderly, with six items related to physical function, four items related to psychosocial function, and two items associated with pain and discomfort. This questionnaire was, for the first time, translated into Persian by Nader Navabi in 2013 [35]. Each question in this section has five possible answers (always, often, sometimes, rarely, and never). The rating of each question varies from at least 1 for “always” up to a maximum of 5 for “never” [27]. The minimum score of this part is 12 and the maximum score is 60. Therefore, a lower overall score is associated with weaker oral health related quality of life, while a higher total score means a better oral health related quality of life.

#### Adult learning theory questionnaire

The third part of the questionnaire consisted of eight researcher-made questions about adult learning theory and the assessment of its effectiveness (Additional file 1). The motivation to learn, experience of learning, readiness to learn, the importance of oral health, willingness to learn, and the need to learn are the contents of the eight mentioned questions. Each of the eight questions in this section also has five possible answers (very high, high, moderate, low, and not at all). The rating of each question ranges from at least 1 for the “not at all” response up to a maximum of 5 for the “very high” answer. The minimum score for this part is 8 and the maximum score is 40. Thus, a higher overall score obtained from this section shows better effectiveness of the theory associated with the oral health related quality of life.

### Validity and reliability

Content validity was used to assess the validity of the questionnaires. Ten experts in the field of Health Education and Oral Health calculated and confirmed the content validity of the questionnaire. The content validity ratio (CVR) and the content validity index (CVI) were equal to 1. Cronbach’s alpha coefficient for the questionnaire items was 0.88, considered as desirable.

### Ethics

This study was approved by the Ethics Committee of Ahvaz Jundishapur University of Medical Sciences (code of IR.AJUMS.REC.1396.315). To consider ethical principles, at the end of this study, participants in the control group were also given adequate education about their oral health care.

### Statistical analysis

Data were analyzed using descriptive statistics (mean and standard deviation) and inferential statistics (t-test, paired t test, chi-squared test and Fischer’s exact test) via SPSS23 (SPSS, Inc., Chicago, IL, USA) software at a significance level of less than 0.05. To compare the effectiveness of education, and due to the normal distribution of oral health related quality of life and the effectiveness of adult learning theory in the two groups, independent t-test was used to compare the change in the overall scores of the two variables prior to and following the treatment; paired t test was further used for intra-group comparison. The distribution of all three variables (total score of physical dimension, psychosocial dimension, and pain-discomfort dimension) was normal in both control and experimental groups. Therefore, independent t-test was used to compare the changes in the score of all three dimensions before and after intervention, and paired t-test was used for intra-group comparison.

## Results

Three subjects in the experimental group were excluded because they did not participate in educational sessions, as were three in the control group because of their cell phone being turned off or for moving from the study area. Finally, the data of 86 people was available for analysis.

**Fig. 1:**
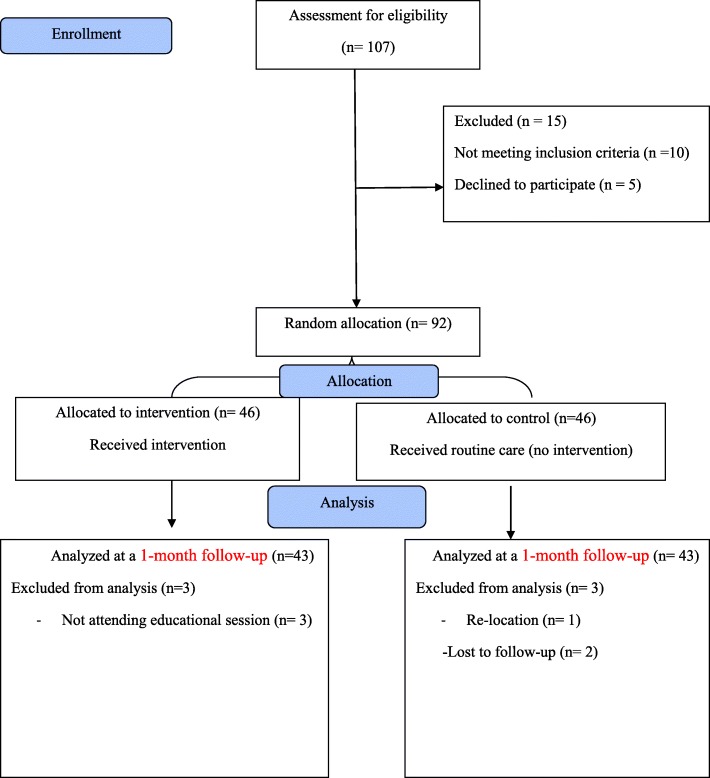
Flow diagram of the study participants

Figure 1 shows the flow diagram of the study participants. The demographic findings of the present study showed that the mean and SD for the participant’s age was 63.35 ± 4.96 years. No significant difference was observed between the two groups in terms of demographic variables prior to the intervention (*P* > 0.05). Table 1 shows the consistency of the two groups in terms of demographic factors affecting the research, including age, gender, academic degree, occupation and marital status.

**Table 1:** Baseline Comparison of the experimental and control groups in terms of demographic variables

Group	All	Intervention (*N* = 43)		Control (*N* = 43)		
		Mean (SD)	Number (%)	Mean (SD)	Number (%)	*P*-value
Age	63.35 (4.96)	63.12 (4.93)		63.95 (5.21)		❖ 0.45
Sex						● 0.66
Female	38 (41.3)		18 (41.9)		20 (46.5)	
Male	54 (58.7)		25 (58.1)		23 (53.5)	
Academic level						○ 0.23
Elementary school	19 (20.7)		12 (27.9)		6 (14.0)	
Intermediate school	12 (13)		4 (9.3)		7 (16.3)	
High school	1 (1.1)		1 (2.3)		0 (0.0)	
Diploma	34 (37)		15 (34.9)		17 (39.5)	
University degree	21 (22.8)		7 (16.3)		12 (27.9)	
Illiterate	5 (5.4)		4 (9.3)		1 (2.3)	
Job						○ 0.29
Worker	3 (3.3)		1 (2.3)		1 (2.3)	
Employee	5 (5.4)		4 (9.3)		1 (2.3)	
Self-employed	17 (18.5)		7 (16.3)		6 (14.0)	
Retired	40 (43.5)		17 (39.5)		22 (51.2)	
Housewife	27 (29.3)		14 (32.6)		13 (30.2)	
Marital status						○ 0.27
Married	79 (85.9)		38 (88.4)		35 (81.4)	
Single	1 (1.1)		0 (0.0)		1 (2.3)	
Widowed	12 (13)		5 (11.6)		7 (16.3)	

❖ Independent t test

● Chi square test

○ Fischer’s exact test

Table 2 Comparisons of the mean score of oral health related quality of life and the effectiveness of adult learning theory prior to and following educational intervention in the experimental and control groups.

**Table 2:** Comparison of the oral health related quality of life and the effectiveness of adult learning theory among the elderly over the study period

Variable	Time	Intervention group (SD) (*N*= 43)	Control group (SD) (*N*= 43)	*P*-value
Overall score of oral health related quality of life	Baseline	48.39 (6.35)	49.74 (6.36)	❖ 0.32
	Follow-up	54.37 (3.15)	49.69 (6.27)	❖ <0.001
	Score change (post test- pre test)	5.98 (4.06)	-0.05 (1.58)	❖ <0.001
	*P*-value	● <0.001	● 0.84	❖ <0.001
Overall score of the effectiveness of adult learning theory	Baseline	34.09 (5.66)	34.62 (5.44)	❖ 0.65
	Follow-up	2.93) 37.62)	5.49 (34.95)	❖ <0.001
	Score change (post test- pre test)	3.53 (3.36)	0.32 (1.04)	❖ <0.001
	*P*-value	● <0.001	● 0.04	❖ <0.001

❖ independent t test

● paired t test

Table 3 compares the experimental and control groups in terms of the overall score of oral health related quality of life associated with physical, psycho-social, and pain-discomfort dimensions before and after educational intervention.

**Table 3:** Comparisons of the mean scores of physical, psychosocial, and pain-discomfort dimensions among the elderly over the study period

Variable	Time	Intervention group (SD) (*N* = 43)	Control group (SD) (*N* = 43)	*P*-value
Physical dimension score	Baseline	23.39 (4.05)	23.76)4.19(	❖ 0.67
	Follow-up	26.97 (2.12)	23.81 (4.21)	❖ <0.001
	Score change	3.58 (2.38)	0.04 (0.81)	❖ <0.001
	*P*-value	● <0.001	● 0.71	❖ <0.001
Psycho-social dimension	Baseline	15.74 (3.23)	16.44 (3.23)	❖ 0.32
	Follow-up	1.74) 18.41)	3.31) 16.39)	❖ 0.001
	Score change	2.67 (2.008)	−0.04 (0.75)	❖ <0.001
	*P*-value	● <0.001	● 0.68	❖ 0.001
Pain-discomfort dimension	Baseline	3.39) 15.93)	2.88) 16.48)	❖ 0.29
	Follow-up	1.35) 19.27)	2.56) 15.93)	❖ <0.001
	Score change	3.34 (2.69)	−0.55 (1.09)	❖ <0.001
	*P*-value	● <0.001	● 0.03	❖ <0.001

❖ independent t test

● paired t test

## Discussion

The purpose of this study was to determine the effectiveness of a theory-centered educational program on oral health related quality of life of elderly persons referred to the dental clinic of Ahvaz Jundishapur University of Medical Sciences, Iran. The findings of this research indicate the positive impact of education based on adult learning theory on the oral health related quality of life of the elderly and the effectiveness of the theory in their training.

A few studies have examined the effect of educational intervention on the life quality of the elderly [36, 37]. However, no research has examined the effect of educational intervention on the oral health related quality of life among elderly persons; therefore, there is no study to compare with the findings of this study or previous studies in this field. The study of Mazloomymahmoodabad et al., showed Education Based on Precede-Proceed quality of life score in experimental as compared to control groups following an intervention [38]. Because oral health is an aspect of an individual’s quality of life [39, 40], it was expected that educational interventions would increase the mean total score of quality of life and the average score of different dimensions of quality of life in the elderly, according to a study conducted on the life quality of the elderly [41]. In the present study, the significant difference between the two groups following the educational intervention is a good indicator of the effect of educational intervention on improving the oral health related quality of life of the elderly. These findings are consistent with the results of the above-mentioned studies. However, Matin et al. found that despite the improvement in the quality of life scores after intervention for an experimental group, there was no significant difference concerning the quality of life scores prior to and after intervention between the two groups [42], which is not consistent with the results of the present study; the reason for such inconsistency is possibly the difference in the educational practices of the two studies; the teaching methods employed in the Matin study were lecture, group discussion, and question and answer, while in the present study, in addition to a group discussion, individual teaching methods and educational text messages were incorporated.

The present research showed that although the mean score of the effectiveness of adult learning theory in the control group slightly increased after the intervention, the average increase in the overall score in the experimental group was much higher, meaning that the theory-driven education was successful in improving the oral health education of the elderly. Because there has been no research on the effectiveness of adult learning theory, it is not possible to compare the findings of this study with other studies. However, most studies done on the impact of educational interventions on the basis of different theories, models, and curricula have proved such interventions to be effective in improving the quality of life of the older population [43]. A study by Sabet Ghadam et al., showed that a Face-to-Face teaching method was effective in increasing the mean scores of quality of life [44]. Given the above-mentioned issues, the application of various theories, models, and educational programs as well as employing different educational methods might be effective in ameliorating the oral health related quality of life problems of older persons and for improving their oral health.

Regarding the effect of educational intervention on the physical dimension of oral health related quality of life of the elderly, the findings of this study showed the positive impacts of education on the mean score of the mentioned dimension. The results of Sabet Ghadam et al. showed that the mean score of physical health problems of Hemodialysis Patients significantly increased following intervention [44], which is in agreement with this study. It is noteworthy that the average score of the physical aspect of oral health related quality of life of the elderly was higher than the other two dimensions after intervention (3.58 points). This is probably due to the importance of eating all types of foods, convenient munching, swallowing, and speaking clearly for the elderly, as oral health problems can affect the mental, psychological, and social life of people. Accordingly, older people are more likely to primarily cure their oral health problems.

The findings of this study indicated that educational intervention had a positive effect on the mean score of the psychosocial dimension of oral health related quality of life. In line with the present research, Pramesona et al. observed an improvement in the mean score of depression following their post test, a difference which was statistically significant [45]. Findings of Dashti et al. showed that a motivational interviewing method was effective in improving the mental health of patients with multiple sclerosis [46]. In the present study, one of the reasons for the improvement in the mean score of the psychosocial dimension of oral health related quality of life of the elderly is probably the individual education based on motivational interviewing at the beginning of the study. The group discussion method has further been helpful in achieving this result, as subjects were able to discuss their opinions, beliefs and experiences on a subject in common, did not feel alone, and hence were mentally more comfortable.

Education had a positive impact on the mean score of the pain-discomfort dimension of oral health related quality of life in the present research. It should be noted that in the control group, the difference between the scores before and after the educational intervention was statistically significant. In terms of the pain-discomfort dimension, the control group was worse in the post-test stage compared with the pre-test stage. Perhaps, one of the reasons is that in addition to general and routine educational programs in dental clinics, there is a need for specialized and targeted educational programs about oral health; in the present study the control group did not receive any specialized education about oral health. The findings of Moreira et al., suggested that neck pain was reduced in two experimental groups following intervention [47], which is consistent with the results of this study. The findings of Aghajani et al., who studyed the effects of self-care education on the quality of life of patients with primary hypertension and compared a lecture and educational package, showed no statistically significant difference in the mean score of pain between the two groups before and after the intervention [48], which is different from the results of the present study, probably because in this study, based on the questions of the participants and the diagnosis of a doctor, none of the subjects had an underlying systemic disease, but the study population in Aghajani et al. study suffered from hypertension.

### The strengths of the study

This research designed a standard questionnaire about the effectiveness of adult learning theory, which can help other researchers who work on adult learning.

### Study limitations

The volunteering of the participants may indicate their motivation, which would act as an intermediary variable not controlled in this study.

The findings of this study cannot be generalized to all elderly, as people from higher socio-economic classes do not refer to the dental clinics of the faculty of dentistry, which is a governmental clinic.

Because of the exclusion criteria, the participants were a group of healthy elderly not a general elderly population, therefore, it is not possible to generalize the results of the study to other groups of people with a systematic illness.

Finally, in this study the oral condition before and after the intervention was not assessed. Because assessing objective measures of the oral condition could provide the researchers with more precise results, we recommend both clinical and subjective variables on health status to be assessed in future research.

## Conclusion

In general, the results of this study showed a significant difference between the mean score of oral health related quality of life, the overall score of the effectiveness of adult learning theory, physical score, psychosocial score, and pain score following the completion of training programs in the experimental group, indicating the positive impact of the training program. Obviously, therapeutic interventions along with oral health education programs can improve oral health related quality of life in the elderly.

## Supplementary information

**Additional file 1.** The researcher-made questions about adult learning theory and the assessment of its effectiveness.

## Data Availability

Upon request, we can offer onsite access to the data analyzed at Ahvaz Jundishapur University of Medical Sciences, Ahvaz, Iran. Dr. Araban should be contacted.

## Abbreviations

CVI: Content Validity Index
CVR: Content Validity Ratio

## References

[1] Khan ZA, Singh C, Khan T (2018). Correlates of physical disability in the elderly population of rural North India (Haryana). J Fam Community Med.

[2] Petersen PE, Yamamoto T (2005). Improving the oral health of older people: the approach of the WHO global Oral health Programme. Community Dent Oral Epidemiol.

[3] Gamage MWK, Hewage C, Pathirana KD (2018). Effect of cognitive and executive functions on perception of quality of life of cognitively normal elderly people dwelling in residential aged care facilities in Sri Lanka. BMC Geriatr.

[4] Cwirlej-Sozanska AB, Sozanski B, Wisniowska-Szurlej A, Wilmowska-Pietruszynska A (2018). Quality of life and related factors among older people living in rural areas in South-Eastern Poland. Annals of agricultural and environmental medicine : AAEM.

[5] Stewart Williams J, Norstrom F, Ng N (2017). Disability and ageing in China and India - decomposing the effects of gender and residence. Results from the WHO study on global AGEing and adult health (SAGE). BMC Geriatr.

[6] Yen YY, Lee HE, Wu YM, Lan SJ, Wang WC, Du JK (2015). Impact of removable dentures on oral health-related quality of life among elderly adults in Taiwan. BMC Oral Health.

[7] Aghamohamadi S, Hajinabi K, Jahangiri K, Asl IM, Dehnavieh R (2018). Population and mortality profile in the Islamic Republic of Iran, 2006–2035. Eastern Mediterranean health journal = La revue de sante de la Mediterranee orientale = al-Majallah al-sihhiyah li-sharq al-mutawassit.

[8] Kossioni AE, Hajto-Bryk J, Maggi S, McKenna G, Petrovic M, Roller-Wirnsberger RE (2018). An expert opinion from the European College of Gerodontology and the European geriatric medicine society: European policy recommendations on Oral health in older adults. J Am Geriatr Soc.

[9] Mohamed N, Saddki N, Yusoff A, Mat JA (2017). Association among oral symptoms, oral health-related quality of life, and health-related quality of life in a sample of adults living with HIV/AIDS in Malaysia. BMC Oral Health.

[10] Soares GB, Garbin CAS, Rovida TAS, Garbin AJÍ (2014). Oral health associated with quality of life of people living with HIV/AIDS in Brazil. Health Qual Life Outcomes.

[11] Talarska D, Tobis S, Kotkowiak M, Strugała M, Stanisławska J, Wieczorowska-Tobis K (2018). Determinants of quality of life and the need for support for the elderly with good physical and mental functioning. Medical science monitor : international medical journal of experimental and clinical research.

[12] Kim EJ, Jung SW, Kim YE, Go DS, Yoon SJ (2018). Assessing the impact of aging on burden of disease. Iran J Public Health.

[13] Haag DG, Peres KG, Balasubramanian M, Brennan DS (2017). Oral conditions and health-related quality of life: a systematic review. J Dent Res.

[14] Kundapur V, Hegde R, Shetty M, Mankar S, Hilal M, Prasad AH (2017). Effect of loss of teeth and its association with general quality of life using geriatric Oral health assessment index (Gohai) among older individuals residing in rural areas. Int J Biomed Sci.

[15] Nguyen HTT, Moir MP, Nguyen TX, Vu AP, Luong LH, Nguyen TN (2018). Health-related quality of life in elderly diabetic outpatients in Vietnam. Patient Prefer Adherence.

[16] Sischo L, Broder HL (2011). Oral health-related quality of life: what, why, how, and future implications. J Dent Res.

[17] Mack F, Schwahn C, Feine JS, Mundt T, Bernhardt O, John U (2005). The impact of tooth loss on general health related to quality of life among elderly Pomeranians: results from the study of health in Pomerania (SHIP-O). Int J Prosthodont.

[18] Cornejo M, Perez G, de Lima KC, Casals-Peidro E, Borrell C (2013). Oral health-related quality of life in institutionalized elderly in Barcelona (Spain). Med Oral Patol Oral Cir Bucal.

[19] Kidd T, Carey N, Mold F, Westwood S, Miklaucich M, Konstantara E (2017). A systematic review of the effectiveness of self-management interventions in people with multiple sclerosis at improving depression, anxiety and quality of life. PLoS One.

[20] Davies N (2011). Promoting healthy ageing: the importance of lifestyle. Nursing standard (Royal College of Nursing (Great Britain): 1987).

[21] Meng K, Musekamp G, Schuler M, Seekatz B, Glatz J, Karger G (2016). The impact of a self-management patient education program for patients with chronic heart failure undergoing inpatient cardiac rehabilitation. Patient Educ Couns.

[22] Cambon J, Cordier T, Munnich EL, Renda A, Kapur B, Hoxhaj S (2018). Effects of educational messaging on urgent and emergent care-seeking behaviors among publicly insured populations. American health & drug benefits.

[23] Jasemzadeh M, Khafaie MA, Jaafarzadeh N, Araban M (2018). Effectiveness of a theory-based mobile phone text message intervention for improving protective behaviors of pregnant women against air pollution: a randomized controlled trial. Environ Sci Pollut Res Int.

[24] Gallagher G, Bell A (2016). Combining adult learning theory with occupational therapy intervention for bladder and bowel management after spinal cord injury: a case report. Occupational therapy in health care.

[25] Nourolahi T, Ghaemi Z, Goodarzi HM, Naeneeni O, Jafari S, Ghaderi S, et al. 1390 national census of population and housing.

[26] Social gerontology in developing countries. A report by participants in the International Short Term Course on Social Gerontology organised by the International Institute on Aging (United Nations, Malta), 17–29 February 1992. Bold : quarterly journal of the International Institute on Aging (United Nations - Malta). 1992;2(3):15–7. 12319350

[27] Atchison KA, Dolan TA (1990). Development of the geriatric Oral health assessment index. J Dent Educ.

[28] Hekmatpou D, Shamsi M, Zamani M (2013). The effect of a healthy lifestyle program on the elderly's health in Arak. Indian J Med Sci.

[29] Villarejo A., Puertas-Martín V. (2011). Usefulness of short tests in dementia screening. Neurología (English Edition).

[30] Allain TJ, Wilson AO, Gomo ZA, Adamchak DJ, Matenga JA (1996). Abbreviated mental test (AMT) in the elderly: shortcoming of an adapted AMT in Zimbabwe. Cent Afr J Med.

[31] Brown V (2018). Infusing adult education principles into a health insurance literacy program. Health Promot Pract.

[32] Lewis SK, Thompson P (2017). Application of adult learning theory to physician assistant education. The journal of physician assistant education : the official journal of the Physician Assistant Education Association.

[33] Lester Richard T, Ritvo Paul, Mills Edward J, Kariri Antony, Karanja Sarah, Chung Michael H, Jack William, Habyarimana James, Sadatsafavi Mohsen, Najafzadeh Mehdi, Marra Carlo A, Estambale Benson, Ngugi Elizabeth, Ball T Blake, Thabane Lehana, Gelmon Lawrence J, Kimani Joshua, Ackers Marta, Plummer Francis A (2010). Effects of a mobile phone short message service on antiretroviral treatment adherence in Kenya (WelTel Kenya1): a randomised trial. The Lancet.

[34] Rashid P (2017). Surgical education and adult learning: Integrating theory into practice. F1000Research.

[35] Navabi N, Salahi S, SHariatmadar ahmadi A (2012). Assessment of Oral Health Assessment Index (GOHAI) Validity in Iranian Elderly Population. Journal of research in dental sciences.

[36] Sun YQ, Jiang AL, Chen SM, Li H, Xing HY, Wang F (2017). Quality of life and self-care in elderly patients with cardiovascular diseases: the effect of a traditional Chinese medicine health educational intervention. Applied nursing research : ANR.

[37] Chafjiri RT, Shirinkam F, Karimi H (2018). Investigating the effect of education on health-promoting lifestyle among the elderly of Ramsar in 2017. Journal of family medicine and primary care.

[38] Mazloomymahmoodabad S, Masoudy G, Fallahzadeh H, Jalili Z (2014). Education based on precede-proceed on quality of life in elderly. Global J Health Sci.

[39] Schiergens TS, Hoffmann V, Schobel TN, Englert GH, Kreis ME, Thasler WE (2017). Long-term quality of life of patients with permanent end ileostomy: results of a Nationwide cross-sectional survey. Dis Colon Rectum.

[40] Banerjee R, Chahande J, Banerjee S, Radke U (2018). Evaluation of relationship between nutritional status and oral health related quality of life in complete denture wearers. Indian journal of dental research: official publication of Indian Society for Dental Research.

[41] Wang Q, Dong L, Jian Z, Tang X (2017). Effectiveness of a PRECEDE-based education intervention on quality of life in elderly patients with chronic heart failure. BMC Cardiovasc Disord.

[42] Matin H, Rastgarimehr B, Afkari ME, Solhi M, Taghdisi MH, Mansourian M (2014). Relationship between the educational stage of precede model and quality of LIFE improvement in the elderly affiliated with TEHRAN culture house for the aged. Iranian Journal of Diabetes and Lipid Disorders.

[43] Barnes AJ, Xu H, Tseng CH, Ang A, Tallen L, Moore AA (2016). The effect of a patient-provider educational intervention to reduce at-risk drinking on changes in health and health-related quality of life among older adults: the project SHARE study. J Subst Abus Treat.

[44] Ghadam MS, Poorgholami F, Badiyepeymaie Jahromi Z, Parandavar N, Kalani N, Rahmanian E (2015). Effect of self-care education by face-to-face method on the quality of life in hemodialysis patients (relying on Ferrans and powers questionnaire). Global J Health Sci.

[45] Pramesona BA, Taneepanichskul S (2018). The effect of religious intervention on depressive symptoms and quality of life among Indonesian elderly in nursing homes: a quasi-experimental study. Clin Interv Aging.

[46] Dashti A, Yousefi H, Maghsoudi J, Etemadifar M (2016). The effects of motivational interviewing on health promoting behaviors of patients with multiple sclerosis. Iran J Nurs Midwifery Res.

[47] de Araujo Freitas Moreira KL, Abalos-Medina GM, Villaverde-Gutierrez C, Gomes de Lucena NM, Belmont Correia de Oliveira A, Perez-Marmol JM (2018). Effectiveness of two home ergonomic programs in reducing pain and enhancing quality of life in informal caregivers of post-stroke patients: a pilot randomized controlled clinical trial. Disabil Health J.

[48] Aghajani M, Mirbagher Ajorpaz N, Kafaei Atrian M, Raofi Z, Abedi F, Naeimi Vartoni S (2013). Effect of self - care education on quality of life in patients with primary hypertension: comparing lecture and educational package. Nursing and midwifery studies.

