# Supplementary material for: Improvement in oral health related quality of life among the elderly: a randomized controlled trial
Source: Biopsychosoc Med. 2019 Dec 5;13:31. doi: 10.1186/s13030-019-0170-3 (PMC6902433; doi:10.1186/s13030-019-0170-3)
Supplement: Supplementary file 1 — Additional file 1. The researcher-made questions about adult learning theory and the assessment of its effectiveness. [file 13030_2019_170_MOESM1_ESM.doc]

| **The questions of measuring the effectiveness of the theory (Adult learning theory)** | | **Very much** | **much** | **moderate** | **low** | | **never** |
| --- | --- | --- | --- | --- | --- | --- | --- |
|  | As an adult, how much do you have motivation to maintain your oral health independently (without dependence on others)? |  |  |  |  | |  |
|  | How much do you want to use your previous experience to keep your teeth healthy? |  |  |  |  | |  |
|  | How much do you have readiness to keep your teeth healthy? |  |  |  |  | |  |
|  | How important is your oral health to you? |  |  |  |  | |  |
|  | How much do you want to learn about oral health? |  |  |  |  | |  |
|  | How much do you want to know why you should learn about your oral health? |  |  |  |  | |  |
|  | How much do you think learning about maintaining your oral health and your teeth is urgently needed? |  |  |  |  | |  |
|  | How much do you feel the motive for your oral and dental health? |  |  |  |  |  | |

The forward only version in English

| **سوالات سنجش میزان اثربخشی تئوری**  **(تئوری آموزش بزرگسالان)** | | | **بسیار زیاد** | | **زیاد** | | **متوسط** | | **کم** | | **اصلا** |
| --- | --- | --- | --- | --- | --- | --- | --- | --- | --- | --- | --- |
| 1 | به عنوان یک فرد بالغ، چقدر برای حفظ سلامت دهان و دندان های خود انگیزه دارید که به طور مستقل(بدون وابستگی به دیگران) عمل کنید؟ | |  | |  | |  | |  | |  |
| 2 | چقدر تمایل دارید که از تجربه های قبلی خود در جهت حفظ سلامتی دهان و دندان هایتان استفاده کنید؟ | |  | |  | |  | |  | |  |
| 3 | در حال حاضر چقدر برای حفظ سلامتی دهان و دندان هایتان آمادگی دارید؟ | |  | |  | |  | |  | |  |
| 4 | چقدر سلامتی دهان و دندان هایتان برایتان مهم است؟ | |  | |  | |  | |  | |  |
| 5 | چقدر تمایل دارید که در مورد سلامتی دهان و دندان یاد بگیرید؟ | |  | |  | |  | |  | |  |
| 6 | چقدر تمایل دارید که بدانید به چه علت باید در ارتباط با سلامتی دهان و دندان هایتان مطالبی را یاد بگیرید؟ |  | |  | |  | |  | |  | |
| 7 | چقدر فکر می کنید که یادگیری در مورد حفظ و افزایش سطح سلامتی دهان و دندان هایتان نیاز فوری شما می باشد؟ |  | |  | |  | |  | |  | |
| 8 | چقدر در خود انگیزه لازم برای حفظ سلامتی دهان و دندان را احساس می کنید؟ |  | |  | |  | |  | |  | |

In Persian
